# Ethnicity and the surgical management of early invasive breast cancer in over 164 000 women

**DOI:** 10.1002/bjs.11865

**Published:** 2021-05-26

**Authors:** T Gathani, K Chiuri, J Broggio, G Reeves, I Barnes

**Affiliations:** 1 Cancer Epidemiology Unit, Nuffield Department of Population Health, University of Oxford, Oxford, UK; 2 Department of Oncoplastic Breast Surgery, Oxford University Hospitals NHS Foundation Trust, Oxford, UK; 3 National Cancer Registration and Analysis Service, Public Health England, Birmingham, UK

## Abstract

**Background:**

Limited information is available about patterns of surgical management of early breast cancer by ethnicity of women in England, and any potential inequalities in the treatment received for breast cancer.

**Methods:**

National Cancer Registration and Analysis Service data for women diagnosed with early invasive breast cancer (ICD–10 C50) during 2012–2017 were analysed. Multivariable logistic regression was used to estimate odds ratios (ORs) and 95 per cent confidence intervals for the risk of mastectomy *versus* breast‐conserving surgery by ethnicity (black African, black Caribbean, Indian, Pakistani and white), adjusting for age, region, deprivation, year of diagnosis, co‐morbidity and stage at diagnosis.

**Results:**

Data from 164 143 women were included in the analysis. The proportion of women undergoing mastectomy fell by approximately 5 per cent between 2012 and 2017 across all the ethnic groups examined. In unadjusted analyses, each ethnic minority group had a significantly higher odds of mastectomy than white women; however, in the fully adjusted model, there were no significantly increased odds of having mastectomy for women of any ethnic minority group examined. For example, compared with white women, the unadjusted and fully adjusted ORs for mastectomy were 1·14 (95 per cent c.i. 1·05 to 1·20) and 1·04 (0·96 to 1·14) respectively for Indian women, and 1·45 (1·30 to 1·62) and 1·00 (0·89 to 1·13) for black African women. This attenuation in OR by ethnicity was largely due to adjustment for age and stage.

**Conclusion:**

Allowing for different patterns of age and stage at presentation, the surgical management of early breast cancer is similar in all women, regardless of ethnicity.

## Abstracta

**Antecedentes:**

Se dispone de información limitada sobre los patrones de tratamiento quirúrgico del cáncer de mama precoz en función de la etnia de las mujeres en Inglaterra, así como de cualquier potencial desigualdad en el tratamiento recibido para el cáncer de mama.

**Métodos:**

Se analizaron los datos del *National Cancer Registration y Analysis Service* (NCRAS) de 164.143 mujeres diagnosticadas de cáncer de mama invasivo precoz (CIE‐10 C50) durante 2012‐2017. Se utilizó la regresión logística multivariable para estimar la razón de oportunidades (*odds ratio*, OR) y los i.c. del 95% del riesgo de mastectomía *versus* cirugía conservadora de la mama según la etnia (negra africana, negra caribeña, india, pakistaní y blanca), ajustado por edad, región, privación, año de diagnóstico, comorbilidad y estadio en el momento del diagnóstico.

**Resultados:**

La proporción de mujeres sometidas a mastectomía disminuyó en ~ 5% durante 2012 a 2017 en todos los grupos étnicos examinados. En los análisis no ajustados, cada grupo étnico minoritario tenía una probabilidad de riesgo significativamente mayor de mastectomía en comparación con las mujeres blancas; sin embargo, en el modelo totalmente ajustado, no hubo probabilidades significativamente mayores de mastectomía para las mujeres de cualquiera de los grupos minoritarios étnicos examinados. Por ejemplo, en comparación con las mujeres blancas, el OR no ajustado y totalmente ajustado (i.c. del 95%) para la mastectomía fue 1,14 (1,05‐1,20) y 1,04 (0,96‐1,14) para las mujeres indias, y 1,45 (1,30‐1,62) y 1,00 (0,89‐1,13) para las mujeres negras africanas. Esta atenuación en el OR por etnia se debió en gran medida al ajuste por edad y estadio.

**Conclusión:**

Admitiendo diferentes patrones de edad y estadio en el momento del diagnóstico, el tratamiento quirúrgico del cáncer de mama precoz es similar en todas las mujeres, independientemente de su origen étnico.

This large national study examined in detail the patterns in surgical management of early breast cancer in women of different ethnicities. Allowing for different patterns of age and stage at presentation, the surgical management of early breast cancer was similar in all women, regardless of ethnicity.

All treated the same

## Introduction

In the 2011 census in England and Wales^1^, 86 per cent of the population was recorded as white, and 13 per cent as black, Asian or other minority groups. The largest single ethnic minority groups within that non‐white population were Indian, Pakistani, black Caribbean and black African people^1^.

Conducting high‐quality healthcare research with a focus on ethnicity has been challenging for many years. Even though self‐reported ethnicity data have been recorded in the census since 1991 and in Hospital Episode Statistics (HES) from 1995, the quality and completeness of the ethnicity information recorded, until recently, has been variable^2–5^. Improvement in the quality of ethnicity data recorded has been driven by the Equalities Act of 2010, which defined a public sector equality duty, and covers eight protected characteristics, including race^4^^,^^6^.

Nationally, breast cancer outcomes have improved significantly over the past couple of decades, but may not be homogeneous across all groups of women; the overall progression seen may mask differences in smaller groups such as women from ethnic minorities^7^^,^^8^. The National Health Service (NHS) Cancer Strategy, 2015–2020, set out a clear vision for the need to improve cancer survival rates as well as the experience of cancer care across all patient groups, noting poorer outcomes from cancer and experience of cancer care in ethnic minority groups^9^.

The incidence of breast cancer is lower in women from ethnic minorities compared with white women; this may be largely due to differences in lifestyle and reproductive patterns^10^. Some studies^8^^,^^11–16^ have suggested that there are differences in survival from breast cancer between women of different ethnic groups, but the reasons for this are not well explained or consistent. Many of these studies have been conducted in single, ethnically dense regions of the country, such as London, Yorkshire and the West Midlands, and with limited information about treatment. These small historical studies also used crude ethnic groupings, for example white, black and South Asian, which may mask differences between distinct groups of people such as Indians and Pakistanis, or black Caribbean and black Africans.

There is also some evidence to suggest that the rates of cancer surgery among women from ethnic minorities may be different from those in white women. The reasons for any observed variations have not been well evaluated, mainly owing to the small numbers of women from ethnic minorities and limited information on treatment history included in the analyses^13^^,^^16^. Surgery is the mainstay of breast cancer treatment and has the largest impact on survival of all the treatment modalities available. Mastectomy and breast‐conserving surgery are the main operations used. Management is influenced by several factors, including stage at presentation, which in turn is influenced by screening patterns. All of these factors may vary by ethnicity and need to be considered when examining any apparent variation in the surgical treatment of breast cancer in different groups of women.

The National Cancer Registration and Analysis Service (NCRAS) is part of Public Health England, and collates data from over 500 regional and local data sets to provide detailed information about breast cancer, including patient characteristics, treatment and stage at presentation^17^. Using this large national data set, the patterns of surgical treatment of early breast cancer are reported here in relation to ethnicity in just under 165 000 women with breast cancer, and with over 1000 women in each of the ethnic groups examined.

## Methods

Data from the Office of National Statistics 2011 census^1^ were used to examine the age distribution of women in England according to ethnicity.

All cancer registrations for invasive breast cancer (ICD‐10 C50) and *in situ* cancer (ICD‐10 D05) in women in England diagnosed between 1 January 2006 and 31 December 2017 were extracted from the NCRAS^17^. In the NCRAS data for breast cancers registered from 2006 to 2011, ethnicity was missing for 26 per cent of registrations. From 2012 to 2017 the proportion of missing ethnicity data fell to 5·5 per cent and, for this reason, the analyses presented here focus on data relating to women diagnosed with breast cancer during that time. Where ethnicity was recorded, women were assigned to one of the five largest ethnic groups: black African, black Caribbean, Indian, Pakistani or white.

Information on the surgical treatment for breast cancer provided by the NCRAS was extracted from the Cancer Outcomes and Services Dataset and from HES data. The main outcome for the present analysis – surgical procedure – is classified as either mastectomy (OPCS‐4 B27) or breast‐conserving surgery (OPCS‐4 B28). Women who initially underwent breast‐conserving surgery, but subsequently required a mastectomy, were included in the mastectomy group for the purposes of analysis.

Other variables used in the analyses included: age at diagnosis (in 5‐year age bands from age 30–79 years), TNM stage at diagnosis (I–IV), and region of diagnosis (9 regions, representing the regional teams of the English cancer registry). A co‐morbidity score was calculated from HES data in the 18 months before breast cancer diagnosis using the Charlson Index (no co‐morbidity or some co‐morbidity). There are 17 categories in the Charlson Index, including conditions such as cardiovascular disease and respiratory disease, with their defined ICD‐10 codes^18^. Socioeconomic status was measured by the income domain of the index of multiple deprivation score (in quintiles). Information was available on the mode of cancer detection (screen‐detected or interval cancer).

Ethical approval for this study was obtained from North East Tyne and Wear South Research Ethics Committee.

### Statistical analysis

The characteristics of women in the five main ethnic groups were compared. The mode of cancer detection was reported for only 99 065 women aged 50–70 years at diagnosis, as women outside this age range are not invited routinely for population‐based screening by current NHS Breast Screening Programme (NHSBSP) criteria^19^.

The percentage of women undergoing mastectomy was calculated by year of diagnosis and by age at diagnosis. Age was classified into five groups according to opportunity for screening: women aged less than 47 years who are not invited for routine screening; women aged 50–70 years who are invited routinely for screening; women aged 47–49 years and 71–73 years who may have been participants of the AgeX trial^20^ and, as such, had cancer detected through screening; and women aged over 73 years who are not invited for screening but can self‐refer if they wish.

A logistic regression model was used to estimate odds ratios (ORs) and 95 per cent confidence intervals for having a mastectomy *versus* breast‐conserving surgery, by ethnicity. Initial analyses were unadjusted, and then adjustment was made individually for the impact of age at diagnosis, region of diagnosis, deprivation, year of diagnosis, co‐morbidity score and stage at diagnosis on mastectomy risk. The effects of combined adjustment for age and stage at diagnosis were investigated, and finally adjustments were made for all the variables simultaneously. Missing values for any of the adjustment variables were assigned to a separate missing category for that adjustment. Sensitivity analyses were also conducted in a subset of women who had complete information on all the relevant confounders.

The reduction in the likelihood ratio χ^2^ statistic associated with ethnicity in the model after adjustment for each variable was calculated, as a measure of the degree to which confounding by the adjustment variable was likely to explain any observed association between ethnicity and risk of mastectomy^21^.

## Results

**Fig. 1:**
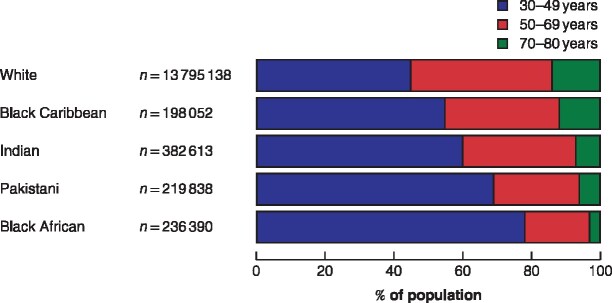
Age distribution of women aged 30–80 years of different ethnicities in England, from 2011 census data

Data from the census showed marked differences in age distribution of women in the English population in the five main ethnic groups (*Fig*. *1*). Less than half of white women were aged under 50 years, compared with almost four of five black African women. Over 40 per cent of white women were aged 50–69 years and therefore eligible for population‐based mammographic screening, compared with one‐third of black Caribbean and Indian women, one‐quarter of Pakistani women and less than one‐fifth of black African women.

Based on NCRAS data, 241 618 women were diagnosed with unilateral invasive breast cancer (ICD‐10 C50) between 2012 and 2017. Some 21 338 women (8·8 per cent) did not have a recorded ethnicity in one of the five groups, and 36 993 (15·3 per cent) were aged less than 30 years or 80 years or over at the time of diagnosis. A further 16 470 women (6·8 per cent) were excluded as they did not have a record of a surgical operation for breast cancer, of whom one‐third (5492) were noted to have metastatic breast cancer (stage IV). Another 2674 women (1·1 per cent) were excluded owing to metastatic disease at presentation and a record of a surgical procedure. The remaining 164 143 women formed the population available for analysis.

Characteristics of the study population are summarized in *Table* *1*. Of the 164 143 women, 157 013 (95·7 per cent) were white; the remaining women were Indian (2701, 1·6 per cent), black Caribbean (1589, 1·0 per cent), Pakistani (1495, 0·9 per cent) or black African (1345, 0·8 per cent). There were significant differences in mean age at breast cancer diagnosis by ethnicity (*P* < 0·001). Women from all ethnic minorities were younger at diagnosis than white women, by a mean of 3–6 years for Indian, black Caribbean and Pakistani women. Black African women were almost a decade younger at diagnosis on average than white women (mean(s.d.) 50·5(11·0) *versus* 59·3(11·2) years). Significant differences were observed for both deprivation and co‐morbidity scores by ethnicity (*P* < 0·001). Women from ethnic minorities were generally more deprived and, except for black African women, in poorer health than white women.

**Table 1.** Characteristics of study population by ethnicity

	% of patients*						
	White	Indian	Black Caribbean	Pakistani	Black African
No. of breast cancers	157 013	2701	1589	1495	1345
Mean(s.d.) age at diagnosis (years)	59·3(11·2)	56·6(11·1)	55·5(11·2)	53·7(11·8)	50·5(11·0)
Most deprived quintile	14·0	20·1	40·9	45·2	44·0
At least one co‐morbidity	24·0	31·1	30·0	41·1	18·4
Stage III disease	9·6	11·9	14·3	15·2	18·8
Screen‐detected cancers in eligible women	58·2	59·6	49·0	48·9	49·6

* Unless indicated otherwise.

Women in all ethnic minority groups were more likely to present with a higher stage of breast cancer than white women, with the highest stage among black African women. Among women who would be eligible for population‐based screening mammography (aged 50–70 years), the proportion of screen‐detected cancers varied significantly by ethnicity. The proportion of screen‐detected cancers in Indian women was similar to that in white women, but the proportion was lower for the other ethnic groups examined.

The crude rate of mastectomy varied significantly by ethnicity; it was lowest in white women (34·8 per cent), and highest in Pakistani (43·6 per cent) and black African (43·7 per cent) women (*Table* *2*). Examination of rates by calendar year of diagnosis and age at diagnosis revealed similar patterns of change over time and associations with age in all ethnic groups. The overall proportion of women undergoing mastectomy fell from 38·1 per cent in 2012 to 31·8 per cent in 2017, and this decline was observed in all five ethnicity groups. A U‐shaped relationship between proportion of women undergoing mastectomy and age at diagnosis was observed in all the ethnic groups. The lowest rates of mastectomy (less than 35 per cent) were seen in women aged 50–70 years who were invited routinely for population‐based screening. In comparison, at least half of women aged below 47 years at diagnosis in all ethnic groups had a mastectomy, and in women aged over 70 at diagnosis, the proportion of mastectomies was higher in all ethnic groups.

**Table 2.** Mastectomy rates by ethnicity, year of diagnosis and age at diagnosis

	**White**	**Indian**	**Black Caribbean**	**Pakistani**	**Black African**
**No. of mastectomies**	54 718 (34·8)	1023 (37·8)	651 (41·0)	652 (43·6)	588 (43·7)
**No. of mastectomies by year of diagnosis**					
2012	9841 (37·8)	169 (42·1)	130 (51·8)	92 (46·9)	93 (49·2)
2013	9919 (37·1)	195 (41·9)	116 (42·0)	95 (41·1)	95 (45·2)
2014	9530 (35·8)	159 (35·3)	124 (43·2)	108 (47·6)	121 (50·2)
2015	8763 (34·1)	166 (36·6)	91 (37·1)	116 (43·1)	85 (37·9)
2016	8554 (32·6)	174 (36·4)	95 (36·1)	118 (42·4)	95 (43·2)
2017	8111 (31·5)	160 (35·3)	95 (35·6)	123 (41·8)	99 (37·9)
**No. of mastectomies by age at diagnosis (years)**					
30–46	10 978 (50·1)	298 (54·9)	165 (53·4)	260 (56·3)	262 (49·9)
47–49 (potential AgeX participant)	5126 (41·5)	69 (34·0)	83 (40·9)	59 (46·1)	78 (44·3)
50–70 (routinely invited by NHSBSP)	27 646 (29·0)	535 (32·3)	296 (34·4)	263 (34·0)	198 (35·4)
71–73 (potential AgeX participant)	3539 (33·8)	36 (30·3)	34 (43·6)	29 (52·7)	19 (55·9)
74–79	7429 (43·7)	85 (47·0)	73 (52·5)	41 (53·9)	31 (60·8)

Values in parentheses are percentages. NHSBSP, National Health Service Breast Screening Programme.

Women from all ethnic minorities had a significantly increased odds of mastectomy compared with white women in unadjusted analyses: OR 1·14 (95 per cent c.i. 1·05 to 1·23) for Indian, 1·30 (1·17 to 1·43) for black Caribbean, 1·45 (1·30 to 1·60) for Pakistani and 1·45 (1·30 to 1·62) for black African women (*Table* *3*). Individual adjustment for age, region, deprivation, year of diagnosis, co‐morbidity and stage all reduced the odds of mastectomy; the largest effects were observed following adjustment for age at diagnosis (64 per cent reduction in likelihood ratio) and stage (90 per cent reduction). After adjustment for age, stage at diagnosis and all other variables, the OR estimates for mastectomy were the same in all ethnic groups: OR 1·04 (0·96 to 1·14) for Indian, 1·11 (1·00 to 1·24) for black Caribbean, 1·03 (0·92 to 1·15) for Pakistani and 1·00 (0·89 to 1·13) for black African women.

**Table 3.** Odds ratios for mastectomy *versus* breast‐conserving surgery in women from ethnic minorities compared with white women

	White	Indian	Black Caribbean	Pakistani	Black African	LR	% reduction in LR
**No. of women**	157 013	2701	1589	1495	1345		
**No. of mastectomies**	54 718	1023	651	652	588		
**Unadjusted odds ratio**	1·00	1·14 (1·05, 1·23)	1·30 (1·17, 1·43)	1·45 (1·30, 1·60)	1·45 (1·30, 1·62)	125	—
**Odds ratio adjusted for**							
Age	1·00	1·11 (1·03, 1·20)	1·20 (1·09, 1·33)	1·28 (1·15, 1·42)	1·16 (1·04, 1·30)	45·3	64
Region	1·00	1·17 (1·08, 1·26)	1·35 (1·22, 1·50)	1·42 (1·28, 1·57)	1·51 (1·36, 1·69)	138·7	–11
Deprivation	1·00	1·12 (1·04, 1·21)	1·24 (1·12, 1·37)	1·38 (1·24, 1·53)	1·38 (1·24, 1·54)	90·8	28
Year of diagnosis	1·00	1·14 (1·06, 1·24)	1·30 (1·17, 1·44)	1·47 (1·32, 1·63)	1·47 (1·32, 1·63)	132·1	–5
Co‐morbidity	1·00	1·14 (1·05, 1·23)	1·29 (1·17, 1·43)	1·44 (1·30, 1·59)	1·46 (1·31, 1·62)	123·2	2
Stage	1·00	1·03 (0·94, 1·12)	1·10 (0·99, 1·23)	1·17 (1·04, 1·30)	1·10 (0·98, 1·23)	13·0	90
Age and stage	1·00	1·01 (0·93, 1·10)	1·06 (0·95, 1·18)	1·07 (0·95, 1·19)	0·94 (0·83, 1·06)	3·5	97
Age, stage and all other variables	1·00	1·04 (0·96, 1·14)	1·11 (1·00, 1·24)	1·03 (0·92, 1·15)	1·00 (0·89, 1·13)	4·6	96

Values in parentheses are 95 per cent confidence intervals. LR, likelihood ratio test statistic. A logistic regression model was used for analysis.

Data were complete for age at diagnosis, region, year of diagnosis and the measure of co‐morbidity using the Charlson Index, whereas information on deprivation was missing for less than 0·1 per cent of the study population, and stage was missing for around 6 per cent. The proportion with missing information on these variables was similar in each ethnic group. When the main analyses were repeated for women with known values for all potential confounders, both the unadjusted and adjusted ORs for mastectomy by ethnicity were similar to those based on the larger data set (data not shown).

## Discussion

These findings show that women in ethnic minority groups are more likely than white women to undergo mastectomy than breast‐conserving surgery for breast cancer, but these differences are largely explained by differences in age and stage at presentation.

The differences in age structures of ethnic populations seen here are a reflection of the temporal patterns of migration into the UK for these groups. Migrant populations are, in general, younger than the white population as most migrants are aged less than 30 years when they enter the UK. Indians and black Caribbeans migrated to the UK in large numbers before the 1970s, and so are now that much older on average; in comparison, black Africans are among the most recent migrants to the UK and four‐fifths of this population is aged under 50 years^22^^,^^23^.

This younger age of ethnic minority populations is important for two reasons with regard to breast cancer. First, it explains the significantly younger mean age at breast cancer diagnosis of women from ethnic minorities compared with white women. It is also of importance when considering potential routes to diagnosis. In the UK, breast cancer is mainly diagnosed as a result of a symptom and subsequent presentation to a breast cancer service in secondary care, or through asymptomatic screening detection through the NHSBSP.

Currently breast cancer screening is offered routinely to women aged 50–70 years in the UK^24^. The primary purpose of population‐based mammographic screening is to improve survival from the disease through earlier detection and downward stage migration, but there are other less highlighted benefits, such as needing less extensive treatment. This is reflected in the present data, which showed a U‐shaped relationship between the proportion of women having mastectomy and age at diagnosis, and the lowest proportion of mastectomies among women aged 50–70 years in all the ethnic groups examined.

In all women, there was a drop in the crude rate of mastectomy of around 5 per cent over the interval studied, as described elsewhere^25^. Breast‐conserving surgery (supplemented with adjuvant radiotherapy) as an alternative to mastectomy has been shown to be a safe treatment choice and explains in part the fall in national mastectomy rates^26^^,^^27^. Although the crude proportion of women having a mastectomy was significantly higher in the ethnic minority groups than among white women, the fall in this proportion over time was similar in women of different ethnicities.

The proportion of women with stage III disease was higher among all the ethnic minority groups than in the white women. Such differences in cancer stage at presentation by ethnicity have been highlighted previously by Public Health England^28^ and other smaller studies^13^^,^^15^. The higher proportion of women with advanced stage disease at presentation among ethnic minority groups may be due in part to a combination of differences in age structures, and/or lack of attendance for screening when offered.

The relationship between ethnicity and breast cancer screening attendance is complex and the existing literature is limited. Variable uptake in screening attendance among women of different ethnicities has been reported, but the data are inconsistent, in part possibly owing to use of crude ethnicity groupings in small studies^28^^,^^29^. Studies using broad classifications, such as ‘south Asians’ to include both Indians and Pakistanis, and ‘blacks’ to include black Caribbeans and black Africans as homogeneous entities, may well mask differences that are likely be pertinent in a sociocultural or religious context. In these data, differences between the smaller groups may well reflect different patterns of health‐seeking behaviour. Among Indian women eligible for routine screening, the proportion of screen‐detected cancers was similar to that among white women, but it was much lower among Pakistani women, suggesting perhaps that Indian women attend for screening in similar patterns to white women but Pakistani women are less likely to attend. Similarly, black Caribbean women had a slightly lower proportion of screen‐detected cancers than black African women. Qualitative research has shown that there are differences in beliefs about breast cancer in these minority communities, and that these beliefs are related to the length of time the migrant population has been present in the host country. These differing beliefs may influence attitudes towards breast health and screening. This in turn can result in delays in presentation to healthcare services, and a lower uptake of screening when offered in certain groups^30–32^.

The patterns of higher rates of deprivation in ethnic minorities are well known^11^^,^^12^^,^^33^^,^^34^ and can affect access to healthcare services, resulting in delayed presentation of disease and a greater likelihood of mastectomy. Higher levels of poorer health of women of Indian, black Caribbean and Pakistani ethnicity have been reported elsewhere^35^. The similar co‐morbidity profile of black African and white women could be explained in part by the younger mean age of the former group, but could also be because the co‐morbidity measure used requires a hospital episode record and this community may not be accessing healthcare to the same extent as other ethnic minority groups. In the present analysis, however, the two most important factors affecting the odds of having a mastectomy were age and stage at diagnosis.

The main strength of this study is the completeness of the ethnicity recording, which was facilitated by legislation introduced in 2010–2011^6^. The impact of this legislation is reflected in the completeness of ethnicity recording in the NCRAS data, which has risen from around 75 per cent to an average over of 94 per cent since 2012. The NCRAS also provides detailed treatment history. This has resulted in large numbers of women with breast cancer in each of the ethnic minority groups examined, enabling a robust and detailed national analysis of the contemporaneous surgical management of early breast cancer in different groups of women.

The study was limited by lack of information on personal characteristics of the women with breast cancer, as the only patient information available related to deprivation and all estimates of co‐morbidity were calculated from hospital records. However, as stage and age were by far the strongest determinants of surgical treatment in the present analysis, the findings are unlikely to have been unduly affected by residual confounding. Information on whether women may have chosen to undergo mastectomy, even if their disease was amenable to breast conservation, is not routinely available. This study used stage of disease as provided by the NCRAS^17^. The possibility of differential misclassification cannot be excluded completely, but the data are used widely and considered to be sufficiently reliable for epidemiological studies.

The results of this large contemporary study suggest that patterns of surgical management are reassuringly similar in women of different ethnic groups, after adjustment for differences in the pattern of presentation of early invasive breast cancer among these groups. Women from ethnic minorities represent a younger group generally, and as such may benefit from targeted public health messaging in their communities with regard to breast health, to encourage more cancer awareness and seeking of early referral to healthcare services, with reassurance that once they engage with the system they are treated similarly. Healthcare professionals working in ethnically dense areas of the country need to be cognisant of the different patterns of presentation of breast cancer in women of different ethnicities, and be encouraged to engage in early referral to secondary care where appropriate.

## Notes

Presented to the Public Health England Cancer Services, Data and Outcomes Conference, Manchester, UK, June 2018, the National Cancer Research Institute Conference, Glasgow, UK, November 2018, the British Breast Group Summer Meeting, Sheffield, UK, June 2019, and the UK Interdisciplinary Breast Cancer Symposium, Birmingham, UK, January 2020; published in abstract form as *Br J Cancer* 2018; **119**: S18 and *Breast Cancer Res Treat* 2020; **180**: 1–49
